# Epiregulin is released from intervertebral disks and induces spontaneous activity in pain pathways

**DOI:** 10.1097/PR9.0000000000000718

**Published:** 2019-03-15

**Authors:** Mette Kongstorp, Tiril Schjølberg, Daniel Pitz Jacobsen, Fred Haugen, Johannes Gjerstad

**Affiliations:** National Institute of Occupational Health, Oslo, Norway

**Keywords:** Disk herniation, Inflammation, Growth factors, Epiregulin, EREG

## Abstract

**Introduction:**

Lumbar radicular pain after disk herniation is associated with local release of many inflammatory molecules from nucleus pulposus (NP) cells leaking out of the intervertebral disk. Here, we have used a rat model to investigate the role of epiregulin (EREG), a member of the epidermal growth factor (EGF) family, in this process.

**Methods:**

A protein immunoassay was chosen to confirm the release of EREG from the NP tissue. Single unit recordings were used to demonstrate the effect of recombinant EREG applied onto the dorsal nerve roots in vivo. Intracellular responses induced by recombinant EREG were studied in cultured dorsal root ganglion (DRG) cells by phosphoprotein assay. Changes in EGF receptor expression induced by NP in the DRG were examined by quantitative polymerase chain reaction.

**Results:**

The protein immunoassay showed that EREG was released from the NP tissue. Moreover, application of EREG onto the spinal dorsal nerve roots induced a decrease in the evoked responses, but an increase in spontaneous activity in the dorsal horn neurons. Interestingly, the EREG activated the phosphatidylinositol 3-kinase (PI3K)/Akt pathway in the DRG, a pathway previously linked to cellular growth, proliferation, and tissue regeneration. An NP-induced upregulation of the EGF receptor HER3 in the DRG was also revealed.

**Conclusion:**

Taken together, the present observations indicate that EREG may induce changes in the DRG and spontaneous activity in the pain pathways. We suggest that EREG signaling may be involved in the pathophysiological process leading to sensory deficits and neuropathic pain in patients after disk herniation.

## 1. Introduction

Lumbar disk herniation causes release of many inflammatory molecules.^9,10,28^ Some of these proinflammatory substances are released from nucleus pulposus (NP) cells,^9^ whereas others may be released from attracted immune cells.^22^ Experimental studies indicate that inflammatory molecules associated with disk herniation may induce sensitization of the dorsal root ganglions (DRG) and pain pathways.^3,4,29^ Moreover, increased levels of circulating cytokines^24^ and growth factors^19^ have been reported after disk herniation in patients. One of these growth factors may be epiregulin (EREG).

Recent data suggest that manipulation with the EREG signaling with blocking one of its receptors, ie, the epidermal growth factor (EGF) receptor (EGFR), may reduce neuropathic pain.^12^ Moreover, processes related to the interaction between EREG and its receptors may be involved in pain processing in temporomandibular disorders.^16^ Taken together, these promising data suggest that interventions targeting EREG signaling may be clinically relevant with regard to future pain treatment. Also, earlier data show that EGFR could interact with many receptors including opioid receptors,^2^ β-adrenergic receptors,^15^ cannabinoid type 1 (CB1), and transient receptor potential cation channel, subfamily V, member 1 (TRPV1) receptors.^31^

Epiregulin is a 46-amino acid protein in the EGF family that binds to the following 4 different receptors: the EGFR and human EGF receptor 2, 3, or 4 (HER2, 3, or 4—in rodents referred to as ErbB2, 3, or 4, respectively). Earlier observations show that EREG induces intracellular signaling through EGFR or HER4. In addition, EREG may bind to HER2 and HER3,^13^ which through heterodimerization with one of the other receptors (a cognate receptor) also promote the intracellular responses, ie, activation of the phosphatidylinositol 3-kinase (PI3K)/Akt pathway and/or the mitogen-activated protein kinase (MAPK) cascade.^32^ Interestingly, both the PI3K/Akt pathway and the MAPK cascade are associated with cellular growth, proliferation, and tissue regeneration.^30,32^

The early release of EREG after injury may be an important step in the successive waves of altered gene expression that promotes long-term changes in neuronal excitability. For example, EREG may trigger expression of other growth factors and cytokines,^7,21^ that in turn through the PI3K/Akt pathway and the MAPK cascade may affect nociceptive activity in the primary afferent nerve fibers^5^ or in the dorsal horn.^25,27^ Also, EREG enhances capsaicin-evoked Ca^2+^ responses in the DRG neurons.^16^ Thus, the aim of this study was to examine the role of EREG in an animal model mimicking the clinical situation after intervertebral disk herniation. Our data suggest that EREG signaling after disk herniation may be associated with sensory abnormalities and pain hypersensitivity.

## 2. Materials and methods

### 2.1. Animals and surgery

Adult inbred female Lewis rats (Harlan Laboratories Inc, Blackthorn, United Kingdom) weighing 190 to 250 g were used in all experiments. The animals were initially sedated with isoflurane gas (Baxter International Inc, Deerfield, IL) for 2 minutes, and anesthetized with intraperitoneal administration of 250 mg/mL urethane (∼2 g/kg bodyweight; Sigma-Aldrich Co, St. Louis, MO). Absence of eye reflexes and foot withdrawal to pinch was considered an indication of sufficient surgical anesthesia. Simplex (80% Vaseline and 20% paraffin) was applied to both eyes to prevent them from drying. The core temperature of the animals was maintained at 36/37°C using a feedback heating pad (homeothermic blanket control unit; Harvard Apparatus Ltd, Kent, United Kingdom). All animal experiments were approved by the Norwegian Animal Research Authority and were performed in confirmation to the laws and regulations controlling experiments and procedures on live animals in Norway.

### 2.2. Protein immunoassay

Harvested NP tissue from the tail vertebrae (of 6 rats) was added to Eppendorf tubes containing HamsF12 medium (Thermo Scientific, Waltham, MA). After 2, 60, 120, and 180 minutes in an incubator at 37°C with 5% CO_2,_ the samples were centrifuged at 4000 rpm for 5 minutes and the supernatant was collected. The supernatant, ie, the conditioned medium, was then stored at −80°C.

A chemiluminescence immunoassay kit (SCB945Ra; Cloud-Clone Corp, Houston, TX) was used to measure EREG in the conditioned medium. A standard with an EREG concentration of 1000 pg/mL was used to make a threefold dilution series. To determine the amount of EREG present in the samples, the chemiluminescence signal was measured with a microplate luminometer (Modulus; Turner Biosystems Inc). The standard dilution series was used to make a standard curve. Unconditioned HamsF12 medium was used as blank in this experiment. The relative light unit value for the unconditioned medium was subtracted from the relative light unit value for each NP sample. The amount of EREG present was calculated by the equation from the standard curve (y = −5E-08x^2^ + 0.0134x + 41.252).

### 2.3. Electrophysiological extracellular single cell recordings

As previously described,^4^ a 5 to 10 mm wide laminectomy was performed on vertebrates Th13-L1, to expose the spinal cord segments L3-S1. A parylene-coated tungsten microelectrode with impedance 2 to 4 MΩ (Frederick Hear & Co., Bowdoin, ME) was lowered into the left spinal dorsal horn using a micromanipulator (MP-285; Sutter Instrument), whereas a reference electrode was placed subcutaneously. The recorded signals were amplified by a headstage and an AC preamplifier, filtered by a band pass filter (Digitimer Ltd, London, United Kingdom) with a half-amplitude cutoff of 500 to 1250 Hz, and digitalized by a 1401 µm interface. All data were stored on a computer using the software spike 2 (4.15; Cambridge Electronic Design, Cambridge, United Kingdom). Spinal cord segments L3-S1 were identified by light tapping on the left paw.

A bipolar silver hook electrode (1.5 mm distance between the hooks) was placed proximal to the main branches of the sciatic nerve for electrical stimulation. A pulse buffer connected to a stimulus isolator unit (NeuroLog System; Digitimer Ltd, United Kingdom) controlled the stimulus intensities. The C-fiber threshold was defined at the beginning of each experiment as the lowest stimulus intensity necessary to evoke a C-fiber response. Every 4th minute throughout the experiment, a single test stimulus (2 ms rectangular pulse, 1.5 × C-fiber threshold) was applied to the sciatic nerve.

Action potentials 50 to 300 ms after each test stimulus were defined as the C-fiber response. The spontaneous activity was defined as action potentials fired between 300 ms after a test stimulus and until the next test stimulus. The C-fiber response on each test stimulus, and the spontaneous activity, was quantified by counting the number of spikes. Six stable C-fiber responses, with variance less than 20%, served as a baseline for the subsequent experiments. Only wide-dynamic-range neurons with a baseline C-fiber response between 5 and 20 spikes were included. To ensure single cell recordings, shape and amplitude of the spike were assessed to discriminate between signals from different cells.

Recombinant EREG (Cloud-Clone Corp, TX) was dissolved in 0.9% NaCl, and diluted to a concentration of 50 µg/mL. One dose of 50 µL EREG or vehicle (0.9% NaCl) was administrated onto the dorsal nerve roots in each experiment. The signal was recorded for 180 minutes after EREG administration (6 rats). Sham operated animals (6 rats), ie, vehicle, served as controls.

### 2.4. Cell culture work

Fresh L3-L5 DRG (of 6 rats) were harvested. The harvested DRG tissues were then dissociated at 37°C with 1.5 mL of papain solution (5000 U) for 30 minutes followed by an equal amount of collagenase/dispase solution (4 mg/mL collagenase and 4.6 mg/mL neutral protease) for 40 minutes (all reagents from Worthington Biochemical Corporation, Lakewood, NJ).

Solutions were removed by centrifugation at 180*g* for 1 minute between the enzymatic disruptions. Next, we added 500 µL of F12/FBS solution (F12 Nut Mix [Ham]; Gibco by Life Technologies, Dublin, Ireland), supplemented with 10% FBS (Gibco by Life Technologies, Grand Island, NY) and 1% penicillin–streptomycin (Gibco Life Technologies, Europe BV, the Netherlands). The tissue was further disrupted by trituration several times through a fire-polished glass Pasteur pipette.

Cell suspension was then applied to a 100-µm cell strainer (Corning Inc, Corning, NY) and washed with DPBS/BSA buffer (DPBS; HyClone Laboratories, Inc, Logan, UT with 0.5g/mL BSA, Sigma-Aldrich Chemie GmbH, Steinheim, Germany). Magnetic labeling and depletion of glia from cell suspension followed the magnetic-activated cell sorting procedure using Anti-GLAST (ACSA-1) MicroBead Kit (Miltenyi Biotec, Bergish Gladback, Germany).

Isolated cells were seeded in 35-mm glass bottom dishes (MatTek Corporation, Ashland, MA) coated with laminin (0.17 mg/mL; Sigma, MO) and poly-d-lysine (0.18 mg/mL; Corning, NY).

Dorsal root ganglion cells were cultivated in Neurobasal-A (NBA) medium at 37°C and 5% CO_2_, where the NBA medium had been supplemented with 2% B27 serum-free supplement (both from Gibco by Life Technologies), 2-mM l-Glutamine (Gibco by Life Technologies), and 5000 U penicillin–streptomycin. After 16 hours, the medium was replaced with NBA medium without B27 supplement. After another 4 hours of incubation, the cells were treated with or without recombinant EREG to a final concentration of 1.0 µg/mL. The exposure lasted for 5 minutes.

The cell lysates were harvested on ice as described in protocol of Bio-Rad Cell Signaling Reagent kit using 300 µL of cell lysis buffer, and stored at −80°C. Finally, the lysates were processed with cell signaling phosphoprotein assays (both from Bio-Rad Laboratories, Inc, Hercules, CA), and analyzed on Bio-Plex MAGPIX (Luminex Corporation, Austin, TX) with Bio-Plex ManagerTM MP Software (Bio-Rad Laboratories, Inc) as described in the supplier's manual.

### 2.5. Gene expression analysis

As previously described,^20^ the expression of the target genes in the DRG and the dorsal horn tissue was examined by quantitative polymerase chain reaction. The animals were euthanized, and the DRG L3, L4, and L5 were harvested and pooled together before gene expression analysis. About 10 mm of the spinal cord was harvested from the laminectomy site (Th13-L1), and then the ipsilateral dorsal horn (left) was rapidly isolated and frozen in liquid nitrogen. Three series of experiments were performed: (1) native (7 rats), (2) control (8 rats), and (3) exposed (8 rats). In the native group, the tissue was harvested immediately after the laminectomy was performed. In the control group, the tissue was harvested 180 minutes after laminectomy. In the exposed group, NP tissue was harvested from 3 to 6 tail vertebrae from a genetically identical donor rat and applied onto the left dorsal nerve roots. After 180 minutes, the NP tissue was removed and the DRG and the dorsal horn were harvested.

Total RNA was isolated form frozen tissue, and reverse transcription of mRNA to cDNA was performed using qScript cDNA synthesis Kit (Quanta Biosciences Inc, Beverly, MA). The gene expression was analyzed by quantitative polymerase chain reaction. All primers for the target genes were designed using Primer Express 3.0.1 (Applied Biosystems, Waltham, MA), and a BLAST search was performed to check for identical sequences in other genes. The primers were designed to span introns in the genomic DNA, to ensure specificity for the desirable mRNA. β-actin was used as a reference gene, and the gene expression of the target genes was normalized to the expression of β-actin. For primer sequences, see Table 1.

**Table 1:**
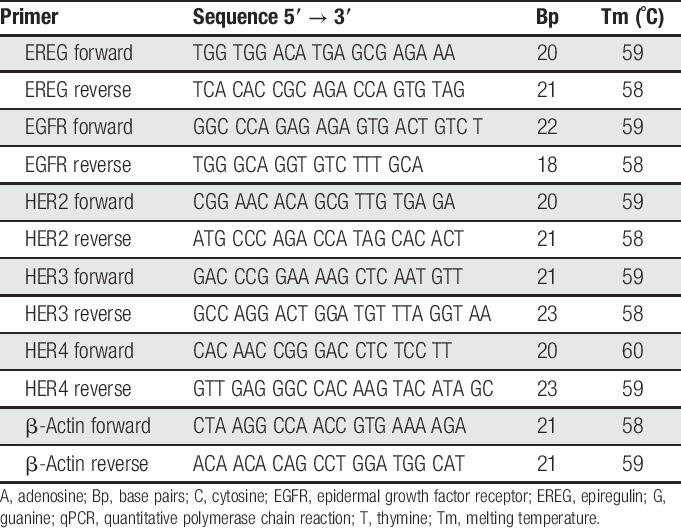
Rat-specific primers used for qPCR.

### 2.6. Statistics

All statistical analyses were performed in SPSS 23 (IBM SPSS Inc, Armonk, NY), and all graphs were created in Sigma plot 12.5 (Systat Software Inc, San Jose, CA). Data are given by examples and by mean ±SEM. A *P*-value below 0.05 was considered significant.

The C-fiber responses (to each electrical test stimulus) were recorded before and until 180 minutes after conditioning. Six stable recordings were set as baseline, and defined as 100%; all other measurements throughout the experiment were given as percentage of baseline. The 6 precondition recordings were converted to 2 baseline values (each comprising 3 recordings), whereas the next 45 postconditioning recordings were converted to 9 after-drug values (each comprising 5 recordings).

The spontaneous activity (between each electrical test stimulus without any stimuli) was also recorded before and until 180 minutes after conditioning. As described above, 6 stable recordings were set as baseline, and the average of the baseline values was subtracted from each recording throughout the experiment. The baseline was then set to 100. Again, baseline recordings were converted to 2 values, and the postconditioning recordings were converted to 9 values (each comprising 5 recordings).

In all in vivo experiments, the effect of EREG on spinal nociceptive signaling over time was compared with the control group by repeated-measures (rm) analysis of variance (ANOVA). When sphericity assumption was not met, a Greenhouse–Geisser correction was applied.

The cell culture data regarding each protein were normalized to total amount of the same protein/β-actin, and analyzed by comparing group means by the Student *t* test. For gene expression analysis, relative quantities were given by comparing the Ct value with the standard curve. Fold-change values were defined by the quantity mean for each sample divided by the quantity of the mean reference gene β-actin. All values were normalized to the mean of the native group. The effect of NP tissue in contact with the dorsal nerve roots were analyzed by comparing groups means by a 1-way ANOVA analysis, with a Tukey's post hoc test.

## 3. Results

**Figure 1.**
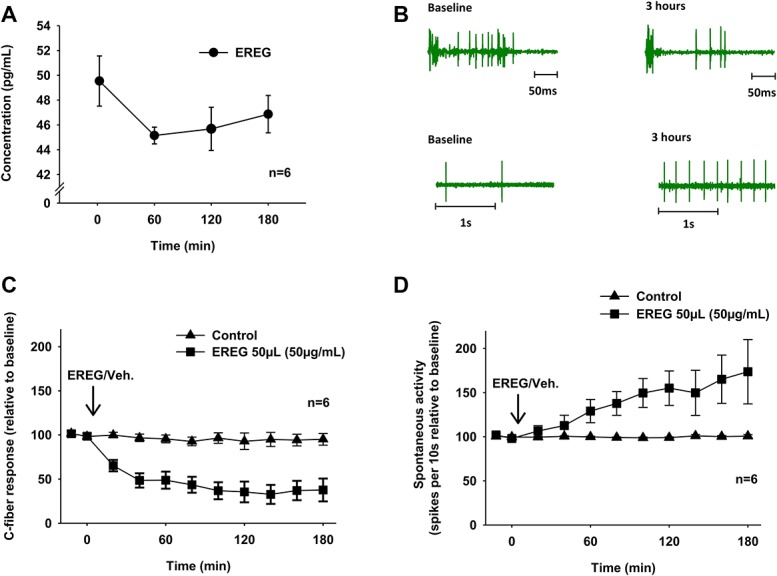
(A) Epiregulin (EREG) levels in medium conditioned by NP relative to EGFR signal in medium without NP (background); 2, 60, 120, and 180 minutes after NP harvesting. (B) Example of single cell recordings at baseline and 180 minutes after administration of EREG. (C) The C-fiber response relative to baseline after administration of 50 µL of EREG (50 µg/mL) or vehicle (Veh.). A decrease in C-fiber response was observed; *P* < 0.001 rmANOVA. (D) The spontaneous activity relative to baseline after 50 μL of EREG (50 μg/mL) or vehicle (Veh.). An increase in spontaneous activity was observed; *P* = 0.026, rmANOVA. EGFR, epidermal growth factor receptor; NP, nucleus pulposus; rmANOVA, repeated-measures analysis of variance.

Analysis of the protein levels in the medium conditioned with NP tissue showed that EREG may be released from the NP cells. The concentration of EREG in the conditioned medium was higher at 2 minutes than at 1, 2, and 3 hours (Fig. 1A). In the dorsal horn, 50 µL of EREG (50 µg/mL) administrated onto the dorsal nerve roots decreased the electrically evoked nociceptive C-fiber response (Fig. 1B top and Fig. 1C; within-subject rmANOVA, *P* < 0.001, EREG group vs control group). By contrast, 50 µL of EREG (50 µg/mL) induced a significant increase in the spontaneous activity in the same nociceptive neurons (Fig. 1B bottom and Fig. 1D; within-subject rmANOVA, *P* = 0.026, EREG group vs control group).

**Figure 2.**
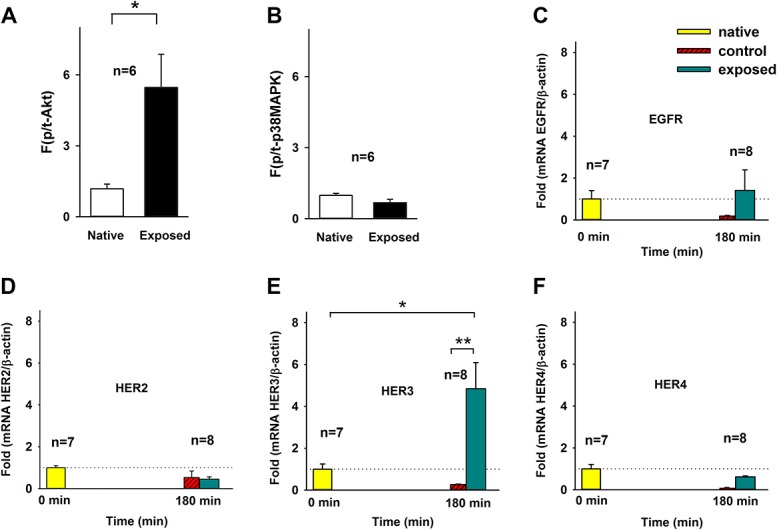
Activation of the intracellular response and changes in gene expression in the DRG. (A and B) Cell signaling phosphoprotein assays (Luminex) of the phosphatidylinositol 3-kinase (PI3K)/Akt pathways and mitogen-activated protein kinase (MAPK) cascade; recombinant EREG vs controls (Veh). * *P* < 0.05 Student *t* test. (C–F) Gene expression assay, qPCR fold expression (normalized to mean of native) of target genes in the 3 groups: native tissue (native), sham operated (control), and NP tissue in contact with the dorsal nerve roots for 180 minutes (exposed). Nucleus pulposus in contact with the dorsal nerve roots for 180 minutes induced a 4.84 ± 1.25-fold upregulation of HER3 in the DRG tissue. No significant changes in the expression of other EGF receptors were observed. * *P* < 0.05, ** *P* < 0.01, 1-way ANOVA, Tukey post hoc test. ANOVA, analysis of variance; DRG, dorsal root ganglion; EGF, epidermal growth factor; EREG, epiregulin; NP, nucleus pulposus; qPCR, quantitative polymerase chain reaction.

In fresh cultured DRG cells, recombinant EREG activated the phosphatidylinositol 3-kinase (PI3K)/Akt pathway (Fig. 2A; *P* = 0.027, Student *t* test, EREG group vs control group), but not the MAPK cascade (Fig. 2B; *P* = 0.053, Student *t* test, EREG group vs control group) in the DRG. In addition, application of NP onto the dorsal nerve roots induced a significant upregulation of one of the EREG receptors, ie, HER3 in the DRG tissue (Figs. 2C–F, 1-way ANOVA, Tukey post hoc test *P* = 0.011, native group vs exposed group).

## 4. Discussion

This study showed that EREG may be secreted from cells found in NP tissue when the NP tissue came in contact with the dorsal nerve roots. Moreover, the effect of EREG applied onto the spinal dorsal nerve roots suggested that EREG signaling through the phosphoinositide 3-kinase (PI3K)/Akt pathway in the DRG may be related to sensory abnormalities and pain hypersensitivity. The local upregulation of HER3 in the DRG cells after application of the NP tissue onto the dorsal nerve roots suggests that leak of NP cells out of the disk may induce changes in the EREG signaling and increased response to further release of EREG.

Earlier studies have shown that EREG may be involved in the development of inflammatory responses.^7^ In addition, EREG may control production of cytokines in macrophages.^26^ In arthritis in mice, EREG induces release of cytokines and other growth factors,^7^ which further may enhance the inflammation.^7,21^ Recruitment of circulating macrophages and release of proinflammatory agents^17,22^ promote nociceptive signaling in primary afferent sensory neurons. Taken together, these findings suggest that EREG released from NP cells may promote local inflammation.

Evidence exists that application of NP onto the dorsal nerve roots affect excitability of dorsal horn neurons^4^ and that NP in contact with the DRG may induce spontaneous neural activity.^29^ In this study, we followed up these findings and showed that EREG applied onto the dorsal nerve roots may influence the neuronal activity. The decrease in C-fiber response and increase in spontaneous activity induced by recombinant EREG suggest that this growth factor clearly affects the nociceptive signaling in the pain pathways.

Actually, the present findings demonstrated that recombinant EREG may have a dual effect on the nerve afferents. In accordance with earlier clinical observations, our data suggest that EREG from NP may be involved in both sensory deficits and persistent pain after disk herniation. Interestingly, recent clinical observations on a patient with neuropathic pain suggest that inhibition of EGFR may reduce neuropathic pain.^12^ In accordance with these clinical observations, our findings show that EREG promote spontaneous activity in nociceptive neurons.

Earlier studies indicate that application of the NP tissue onto the dorsal nerve roots induces a significant reduction in nerve conduction velocity.^11,23^ Moreover, epidural application of NP tissue may result in rapid increase in vascular permeability in spinal nerves.^1^ These physiological changes may lead to abnormal neural signaling underlying sensory deficits. In this study, we show that NP also could release molecules such as EREG. Thus, it is tempting to speculate that EREG contributes to the neuronal changes underlying the decrease in nerve conduction. This might explain the reduced C-fiber response observed after local application of recombinant EREG onto the dorsal nerve roots.

In addition, release of molecules such as EREG from NP could, through intracellular second messenger systems such as the PI3K/Act pathway or MAPKs such as ERK/CREB,^25,27^ influence on the excitability of the pain pathway. Experiments performed on DRG neurons in rats suggest that PI3K may sensitize primary afferent neurons by upregulation of TRPV1.^5^ Thus, signaling through PI3K and ERK may mediate inflammatory changes in primary afferent neurons and subsequent hyperalgesia through TRPV1 sensitization.^33^

Previous studies have shown that inhibition of PI3K activation may reduce phosphorylation and activation of the *N*-methyl-d-aspartate receptor and decreased translocation of α-amino-3-hydroxy-5-methyl-4-isoxazole propionate receptors to the plasma membrane in spinal neurons.^25^ Moreover, intracellular signaling after activation of the HER receptors may lead to activation of several transcription factors including CREB, a protein earlier linked to spinal cord long-term potentiation.^14^ Interestingly, CREB alter gene expression of many genes including the NK1 receptor and BDNF.^8,27^

In this study, we demonstrated that application of NP onto the dorsal nerve roots induced an upregulation of HER3 in the DRG tissue. Interestingly, previous studies have demonstrated that HER3 is up-regulated in the DRG neurons after spinal cord injury.^18^ Hence, leak of NP onto the DRG seems to induce HER expression changes similar to the changes observed after nerve injury. Moreover, HER3 may be activated by neuregulin important for remyelation in the DRG.^6^ However, because only mRNA was measured in this study, our data do not give exact information about the protein expression.

Moreover, the recombinant EREG used in this study to examine the role of the EGFR, HER2, HER3, and HER4 may have unspecific effects. In addition, a mix of molecules, not only EREG, was probably released from the NP in our model. Thus, precisely which molecule that induced the upregulation of HER3 remains to be investigated. Finally, whether or not the increase in the HER3 expression in the DRG really affects the EREG signaling is also dependent on EGFR, HER2, and HER4.

## 5. Conclusion

Disk herniation and leak of NP cells out of the disk may induce a local immune response.^11^ Many proinflammatory molecules, including EREG, may be released in this process. Our results show that EREG can promote spontaneous activity in the nociceptive dorsal horn neurons. Interestingly, EREG seemed to involve activation of the PI3K/Akt pathway in the DRG—a pathway earlier linked to cellular growth, proliferation, and tissue regeneration. The NP also seemed to increase the expression of HER3 in the same DRG cells, which may increase intracellular response to further release of EREG. Taken together, these data suggest that EREG signaling may be involved in the pathophysiological process leading to sensory deficits and neuropathic pain in patients after disk herniation.

## Disclosures

The authors declare no conflicts of interest.
